# Radiotherapy in the management of early breast cancer

**DOI:** 10.1002/jmrs.1

**Published:** 2013-02-03

**Authors:** Wei Wang

**Affiliations:** 1 Westmead Breast Cancer Institute, Westmead HospitalWestmead, New South Wales, Australia; 2 Department of Radiation Oncology, Westmead HospitalNew South Wales, Australia

**Keywords:** Early breast cancer, radiotherapy

## Abstract

Radiotherapy is an indispensible part of the management of all stages of breast cancer. In this article, the common indications for radiotherapy in the management of early breast cancer (stages 0, I, and II) are reviewed, including whole-breast radiotherapy as part of breast-conserving treatment for early invasive breast cancer and pre-invasive disease of ductal carcinoma in situ, post-mastectomy radiotherapy, locoregional radiotherapy, and partial breast irradiation. Key clinical studies that underpin our current practice are discussed briefly.

## Introduction

Radiation was first utilized to treat breast cancer just before the turn of 20th century. In 1896, less than a year after Wilhelm Conrad Röntgen discovered the x-ray, a medical student in Chicago named Emil Grubbe assembled his own x-ray machine and used it to treat a woman with recurrent breast cancer.^1^ Moving forward to today, radiotherapy is a fundamental part of the management of all stages of breast cancer. In this article, the common indications for radiotherapy in the management of early breast cancer (stages 0, I, and II) are reviewed, along with brief introduction of key clinical studies that underpin the current practice.

## Whole-Breast Radiotherapy as Part of Breast-Conserving Treatment for Early Invasive Breast Cancer (Stages I–II)

Treatment options for early breast cancer include breast-conserving treatment and total mastectomy. Breast-conserving treatment consists of a wide local excision which removes breast cancer with a margin of normal breast tissue, sentinel lymph node biopsy, and/or axillary clearance, followed by whole-breast radiotherapy. For most women with early stage breast cancer, breast-conserving treatment is not only feasible but a curative treatment option. Breast-conserving treatment preserves the patient's native breast, and in vast majority of cases, it results in good cosmetic outcome. Breast radiotherapy is well tolerated by women. For most, common side effects such as skin erythema and fatigue are reversible within a few weeks from the completion of radiotherapy. Significant moist desquamation of the skin is uncommon and late complications such as rib fracture, radiation pneumonitis, and second malignancy are rare. In contrast, patients treated by total mastectomy, who wish to restore normal breast shape and contour, need to undertake lengthy and complex breast reconstructive surgery with either breast implant or autologous tissue, either immediately during mastectomy or as a delayed procedure following mastectomy. However, breast-conserving surgery can achieve satisfactory cosmetic result only if adequate amount of breast tissue can be preserved following margin-negative resection of breast cancer. Therefore, patients with large tumour relative to the breast size or patients with diffuse malignant-appearing microcalcification indicating widespread pre-invasive disease of ductal carcinoma in situ (DCIS) are not suitable candidates for breast-conserving treatment. Other contraindication for breast radiotherapy and therefore breast-conserving treatment includes previous moderate- to high-dose radiotherapy to the breast or chest wall, pregnancy, and active connective tissue disease such as scleroderma or systemic lupus erythematosus (NCCN clinical practice guideline in oncology).

A meta-analysis of 3100 women in seven randomized trials comparing mastectomy with breast-conserving surgery plus radiotherapy showed identical overall survival rate at 10 years.^2^ The long-term results of the NSABP-06,^3^ EORTC 10801,^4^ and Milan-I^5^ trials showed no significant difference in distant disease-free survival and overall survival rate for patients treated by breast-conserving treatment and mastectomy after 20-year follow-up. These studies showed that the risk of local recurrence after breast-conserving treatment was slightly higher compared with mastectomy.^6^ However, with better breast imaging to facilitate preoperative planning, meticulous examination of surgical margin and re-excision of any positive or close margins, improved quality of breast radiotherapy, and more effective systemic treatment options, local recurrence rate after breast-conserving treatment in the modern era is much lower compared with previous decades (more modern trial such as the EORTC boost vs. no boost trial,^7^ Canadian OCOG trial^8^ showed lower local recurrence rate at 10 years after breast-conserving treatment, 10.2% and 6.7%, respectively). Furthermore, breast cancer patients are followed up regularly by frequent clinical examinations (3–6 monthly for the first 2 years, and 6–12 monthly thereafter in our institution) and annual breast imaging, and most local recurrence of breast cancer is discovered early and can be readily salvaged by mastectomy.

Radiotherapy to the breast is an essential component of breast-conserving treatment. The Early Breast Cancer Trialists' Collaborative Group (EBCTCG) meta-analysis of randomized controlled trials estimated that local recurrence rate is 29.2% and 46.5% at 10 years for node-negative and node-positive breast cancer treated with breast-conserving surgery alone, and the risk is reduced to 10% and 13.1%, respectively, by the addition of breast radiotherapy.^9^ Despite complete surgical resection of the gross tumour, there is still significant risk that the patient has low-volume residual disease within the breast. Holland et al.^10^ examined mastectomy specimens of 282 cases of invasive breast cancers and found that small foci of disease are present in 63% of cases at 2–4 cm around the main tumour mass. Radiotherapy to the breast can potentially eradicate residual low-volume disease within the breast and thereby significantly improves local control. And the improved control of local disease means there is reduced risk of further distant metastases from the local residual or recurrent disease, and this can lead to a reduction in breast cancer mortality. In the latest EBCTCG meta-analysis of over 10,000 women from 17 randomized trials, the use of radiation after breast conservation reduced breast cancer mortality at 15-year follow-up by 3.3% for women with node-negative breast cancer and 8.5% for women with node-positive disease.^11^ Overall, for every four local recurrences avoided by year 10, approximately one breast cancer death is avoided by year 15.^9^

The target area for post-operative radiotherapy following breast-conserving surgery is the whole breast. The standard radiation dose recommended for whole-breast radiotherapy is 45–50 Gy in 25 fractions, followed by boost radiation therapy to the tumour cavity at a dose of 10–16 Gy in 5–8 fractions. The use of boost radiation therapy to the tumour bed was based on the result of clinical trials (EORTC boost vs. no boost trial,^7^ Lyon boost trial^12^) demonstrating further reduction in local recurrence rate by the addition of boost radiation. Some clinician would recommend the use of boost radiation therapy for all patients, while others are concerned about the worse cosmetic outcome resulted from the boost radiation and therefore reserve it for patients at higher risk of local recurrence. Young age, positive or close margins, high-grade disease, presence of extensive DCIS and, to a lesser extent, the presence of lymphovascular invasion, and lymph node positivity are all factors indicating a higher risk of local recurrence, and the use of boost radiation can potentially reduce the negative impact of these factors.^13^–^15^ A recent analysis of the EORTC boost versus no boost trial showed that for patients younger than 50 years old and in patients with high-grade invasive ductal carcinoma, the boost dose significantly reduced local recurrences from 19.4% to 11.4% (*P* = 0.0046; HR = 0.51) and from 18.9% to 8.6% (*P* = 0.01; HR = 0.42), respectively.^15^

Multiple randomized controlled trials (Canadian OCOG trial,^8^ START A,^16^ and START B^17^) have also shown that hypofractionated radiotherapy, that is, the use of daily fraction of greater than the standard fraction size 1.8–2 Gy, achieved equivalent efficacy and cosmetic outcome compared with the standard fractionation regimen. Therefore, hypofractionation regimen such as 42.5 Gy in 16 fractions (Canadian OCOG trial^15^) or 40 Gy in 15 fractions (START B trial^17^) has now been accepted an alternative standard for whole-breast radiotherapy. The benefit of hypofractionated regimen is that treatment time is now reduced from 5 weeks to approximately 3 weeks, which is more convenient for the patients and spares valuable radiotherapy resource. As the majority of patients who participated in the randomized controlled trials were aged 50 years and older, with breast tumour <5 cm in size, with no axillary lymph node involvement, and did not have systemic chemotherapy, American Society for Radiation Oncology (ASTRO) evidence-based guidelines recommend the use of hypofractionated whole-breast radiotherapy for the above group of patients.^18^

## Whole-Breast Radiotherapy as Part of Breast-Conserving Treatment for DCIS (Stage 0)

With the success of the breast cancer screening programme using screening mammogram, increasingly breast cancer is discovered at the pre-invasive stage of DCIS. DCIS is not a life-threatening disease, because in pure DCIS, the cancer cells are confined within the duct structures of the breast and lack the capacity to invade; hence, there are no lymphatic or distant haematogenous metastases. DCIS, however, has the capacity to recur locally. Approximately half of the recurrences are invasive cancer, and invasive recurrence has the ability to metastasize to distant organs. There have been no randomized controlled trials comparing total mastectomy and breast-conserving treatment for DCIS. While total mastectomy achieves a lower risk of local recurrence compared with breast-conserving treatment, several non-randomized studies showed that there was no significant difference in breast cancer–specific survival or overall survival between the two treatment options.^19^,^20^ Whole-breast radiotherapy significantly improves local control after wide local excision alone and is an important part of breast-conserving treatment for DCIS. At least four randomized controlled trials (NSABP-B17,^21^,^22^ EORTC 10853,^23^,^24^ Swedish DCIS trial,^25^ and UK/ANZ DCIS trial^26^) have shown that the addition of whole-breast radiotherapy to wide local excision significantly reduced the risk of local recurrence, and both the risk of recurrent DCIS and recurrent invasive breast cancer were significantly reduced. The recent meta-analyses of DCIS randomized trials by EBCTCG showed that radiotherapy reduced the absolute 10-year risk of any ipsilateral breast event (i.e., either recurrent DCIS or invasive cancer) by 15.2%, from 28.1% to 12.9% (*P* < 0.00001).^27^

The vast majority of patients enrolled in the randomized controlled trials were treated with standard fractionation regimen (i.e., 50 Gy in 25 fractions) without the addition of boost radiation to the tumour bed. As mentioned previously, in the setting of invasive breast cancer, hypofractionated regimen is equally efficacious when compared with standard fractionation regimen, and the addition for boost radiation to the tumour bed further reduced the risk of local recurrence. It is not clear if these findings also apply to the treatment of DCIS; the currently open TROG 07.01 phase III trial is evaluating the efficacy of hypofractionated whole-breast radiotherapy (42.5 Gy in 16 fractions vs. 50 Gy in 25 fractions) and the addition of boost radiation to the tumour bed (16 Gy in 8 fractions vs. no boost) for patients with DCIS treated by breast-conserving treatment requiring whole-breast radiotherapy.

## Post-mastectomy Radiotherapy for Large and/or Node-Positive Breast Cancer

For patients with regional lymph nodes involved by breast cancer, large breast cancer >5 cm in size, tumour involved surgical margin, the risk of locoregional recurrence is still high despite total mastectomy and axillary clearance. Post-mastectomy radiotherapy is indicated to reduce the risk of locoregional recurrence.^28^ Randomized controlled trials (British Columbia post-mastectomy radiotherapy trial,^29^ DBCG 82b/c^30^,^31^) have shown that for patients at high risk of locoregional recurrence, post-mastectomy radiotherapy not only reduced the risk of locoregional recurrence but also improved the overall survival. There is general acceptance that patients with four or more involved axillary lymph nodes are at high risk of recurrence in the chest wall and regional lymph nodes, and post-mastectomy radiotherapy to these sites is indicated.^28^

However, there is still ongoing debate as to whether post-mastectomy radiotherapy should be routinely recommended to patients with one to three involved lymph nodes. The 2001 guideline from American Society of Clinical Oncology (ASCO) stated that “there is insufficient evidence to make recommendations or suggestions for the routine use of post-mastectomy radiotherapy in patients with T1/2 tumours with one to three positive nodes.”^28^ The ASCO guideline recommendation has since been challenged by several recent studies, including the subgroup analysis of the Danish trial^32^ and the EBCTCG meta-analysis;^7^ all showed significant survival benefit of locoregional radiotherapy for patients with one to three positive axillary nodes. Overgaard et al. argued that post-mastectomy radiotherapy is potentially more important in the intermediate-risk patients with one to three involved lymph nodes, because this group of patients has lower risk of distant metastases compared with patients with four or more involved lymph nodes, and therefore, the improvement in locoregional control as a result of the post-mastectomy radiotherapy will have a greater impact on breast cancer survival.^32^ The current NCCN guideline recommends that clinicians give strong consideration for the use of post-mastectomy radiotherapy for patients with one to three involved lymph nodes. Most radiation oncologists will weigh up important patient, tumour, and treatment factors before making a recommendation. These factors include the age, performance status and comorbidity of the patients, the number of involved axillary lymph nodes, the extent of tumour involvement and presence or absence of major extra-nodal extension of disease, size and grade of the primary tumour, evidence of lymphovascular invasion, hormone receptor and Her 2 receptor status, the adequacy of the total mastectomy and surgical margin status, the adequacy of axillary dissection (at least 10 axillary lymph nodes examined), and if systemic chemotherapy or endocrine therapy is employed.

The target areas for post-mastectomy radiotherapy include the chest wall and/or regional lymph nodes. Typical dose recommended is 50 Gy in 25 fractions. Megavoltage photon radiotherapy commonly used for chest wall radiotherapy is skin sparing. Local recurrence of breast cancer after total mastectomy frequently occurs in the skin or subcutaneous tissue immediately below the skin.^34^ To ensure adequate dose of radiation to the skin and subcutaneous tissue, a layer of tissue equivalent “bolus” is frequently applied to the chest wall, so that the prescribed dose of radiation is delivered to the skin surface and subcutaneous tissue immediately below the skin. There is, however, no international consensus regarding the indications for the use of bolus, thickness of the bolus, or the frequency of its use.^35^

Routine irradiation of internal mammary chain (IMC) lymph nodes during post-mastectomy radiotherapy or locoregional radiotherapy is still considered controversial, given that isolated IMC nodal recurrence of breast cancer is rarely encountered.^36^ And irradiation of IMC lymph nodes significantly increases the dose to the lung and heart (especially for left-sided cases), and this can potentially lead to increased cardiac morbidity and mortality.^37^ Most randomized controlled trials showing a benefit of post-mastectomy radiotherapy, such as the British Columbia and Danish post-mastectomy radiotherapy trial, all included IMC as a target area. A French randomized controlled trial randomly allocated patients to receive IMC irradiation versus not and failed to show a statistically significant benefit for IMC irradiation in terms of overall survival at 10 years (59.55% for no IMC irradiation vs. 62.57% for IMC irradiation, *P* = 0.8762).^38^ However, the criticism is that the trial may be underpowered to detect a small survival difference of 3%. In our institution, lymphoscintigraphy after peri-tumoural injection of radioactive colloid is routinely performed, and mapping to the IMC is detected in approximately 15–20% of cases.^39^ The practice in our institution is to sample the IMC sentinel node when there is evidence of mapping to the IMC, and we would irradiate the IMC if the IMC sentinel node is found to be pathologically involved.

## Locoregional Radiotherapy for Node-Positive Breast Cancer Treated by Breast-Conserving Surgery

For patients treated by breast-conserving surgery, regional nodal irradiation is generally not indicated if axillary lymph nodes are not involved. On the other hand, if there is involvement of more than three axillary lymph nodes, there has been general acceptance that the risk of regional nodal relapse is sufficiently high to justify the addition of regional nodal irradiation in addition to whole-breast radiotherapy.

The recent MA20 trial^33^ evaluated the role of regional nodal irradiation for patients mostly with one to three positive axillary lymph nodes treated by breast-conserving treatment. This trial showed that the addition of regional nodal irradiation to the supraclavicular lymph nodes, apex of axilla, and internal mammary lymph nodes significantly improved 5-year disease-free survival from 84% to 89.7% (*P* = 0.003), and there was also borderline significant improvement of 5-year overall survival from 90.7% to 92.3% (*P* = 0.07). The MA20 trial result is yet to be fully published, but it has the potential to change clinical practice, that is, the recommendation of regional nodal irradiation for all patients with node-positive disease treated by breast-conserving surgery.

## Accelerated Partial Breast Irradiation

In recent years, there has been ongoing interest regarding partial breast irradiation and ongoing debate whether it achieves similar efficacy to whole-breast radiotherapy for some patients. Seventy to eighty percent of local recurrences occur at the sites of the primary tumour.^40^–^42^ This prompted the current interest in partial breast irradiation targeting breast tissue 1–2 cm around the surgical cavity. The potential rationale of partial breast irradiation is that it may achieve similar local control of breast cancer for selected patients, and it has the benefit of limiting radiation to small part of the breast, and thereby reducing the amount of radiation delivered to breast tissue further away, as well as nearby organ of lung and heart. Another potential benefit is that partial breast irradiation is delivered using a much shorter dose fractionation, so treatment is usually completed intraoperatively or over 5 days, which increases the convenience to the patients.

Multiple prospective studies have shown that partial breast irradiation with various techniques can achieve low rate of local recurrence and acceptable toxicity.^43^–^49^ However, several large multi-centre phase III randomized controlled trials, such as NSABP-B34, RAPID trials, comparing the efficacy and toxicity of accelerated partial breast irradiation against conventional whole-breast irradiation are still ongoing. The early results from one of the large randomized controlled trial – TARGIT trial – were recently published, and this trial randomized patients to receive intraoperative radiotherapy using Intrabeam device to the surgical cavity or conventional whole-breast irradiation.^50^ At 4 years, almost equivalent local recurrence rate was found in the two arms (1.2% in the intraoperative radiotherapy group and 0.95% in the whole-breast radiotherapy group, *P* = 0.41), and there was no significant difference in treatment-related toxicity.

Various techniques have been used to achieve partial breast irradiation, ranging from intraoperative radiotherapy, interstitial brachytherapy, mammosite or other balloon catheter brachytherapy system, and external beam radiotherapy using multiple non-coplanar beam arrangement. The dose fractionation regimen used differs depending on the technique employed. For example, the Intrabeam intraoperative radiotherapy device delivers a dose of single fraction of 20 Gy using 50 kV soft x-ray. The typical regimen used for interstitial or mammosite brachytherapy is 34 Gy in 10 fractions, twice daily, over 5 days. Accelerated partial breast irradiation delivered by external beam radiotherapy typically uses a dose fractionation of 38.5 Gy in 10 fractions, twice daily, over 5 days.

Recently, the ASTRO released a consensus guideline,^51^ dividing patients into “suitable group,” “cautionary group,” and “unsuitable group” for accelerated partial breast irradiation. The “suitable group” is patients aged 60 years or older, without BRCA1/2 mutation, with unicentric and clinically unifocal tumour of size less than or equal to 2 cm, excised with negative margin of at least 2 mm, with no evidence of lymphovascular invasion and no evidence of lymph node involvement, oestrogen receptor positive, invasive ductal or other favourable subtypes. Pure DCIS or tumour with extensive intraductal component is not considered in the “suitable group.” Currently, most Australian centres have not adopted the ASTRO consensus guideline, and generally reserve the use of accelerated partial breast irradiation within the context of prospective clinical trials, while waiting for the definitive and longer term results from multiple ongoing phase III randomized controlled trials evaluating the efficacy and toxicity of accelerated partial breast irradiation.

## Conclusion

With improvement in the quality of all modalities of breast cancer treatment – surgery, radiotherapy, and systemic therapy – the locoregional control of early breast cancer has improved significantly. Therefore, increasingly the focus of current research is to minimize the toxicity associated with radiotherapy. Some of the examples are intensity-modulated radiotherapy, which has been shown to reduce the rate of moist desquamation,^52^ and deep inspiration breath hold radiotherapy, which has been shown to reduce the radiation dose to the heart^53^,^54^ and potentially reduce late cardiac morbidity or mortality.

Radiotherapy is already an integral part of the management of early breast cancer, and it has been shown to reduce the risk of locoregional recurrence and improve breast cancer survival. And as more evidence from clinical trials becomes known, the indications of breast radiotherapy will continue to evolve, and the techniques of radiotherapy will be further refined.
